# A Case of Post-COVID Mucormycosis in a Diabetic Patient in Pakistan

**DOI:** 10.7759/cureus.74852

**Published:** 2024-11-30

**Authors:** Bakhtawar Awan, Mohamed Elsaigh, Areej Tariq

**Affiliations:** 1 General and Emergency Surgery Department, Northwick Park Hospital, London, GBR; 2 General and Emergency Surgery Department, Royal Cornwall Hospitals NHS Trust, Truro, GBR; 3 Ophthalmology Department, Shaikh Zayed Hospital, Lahore, PAK

**Keywords:** case report, fungal rhinosinusitis, invasive fungal infections, mucormycosis, opportunistic fungal infections, post-covid-19 mucormycosis, uncontrolled diabetes mellitus

## Abstract

Mucormycosis is a rare but potentially fatal angioinvasive fungal infection, caused by filamentous molds of the order Mucorales, which primarily affects immunocompromised individuals and is characterized by high mortality rates. Diabetes mellitus (DM) is the most common risk factor for mucormycosis. During the COVID-19 pandemic, the number of cases significantly increased. Mucormycosis may present in several clinical forms, but the most common form is the rhinocerebral form. This case report aims to emphasize the severity of mucormycosis in a patient with uncontrolled DM who recently recovered from COVID-19. Furthermore, this study demonstrates the importance of early diagnosis and proper treatment to prevent fatal progression.

A 58-year-old man is presented in our case report; the patient was recently diagnosed with DM and presented to the outpatient department (OPD) with complaints of right eye ptosis and progressively worsening painful vision loss over 10 days. He had been infected with SARS-CoV-2 infection 15 days earlier and was treated at home with systemic corticosteroids and antibiotics. One week after recovery, he experienced spontaneous nasal bleeding, followed by progressive ocular symptoms that culminated in restricted eye movement and complete vision loss in the right eye. Physical examination revealed right eye proptosis and no light perception. Laboratory tests indicated an elevated C-reactive protein (CRP) level of 25.65 mg/dL and a significantly high glycated hemoglobin A1c (HbA1c) level of 16.3%. A tissue biopsy showed inflammatory nasal polyps associated with mucormycosis. The patient was promptly treated with insulin in order to control the elevated HbA1c and amphotericin B to stop the fungal infection and underwent extensive surgical excision.

Our case highlights the severity of mucormycosis in a patient with uncontrolled DM who recently recovered from COVID-19 and shedding light on the importance of controlling the DM in order to reduce the negative impacts on the patient's health. The patient underwent proper treatment with antifungals and aggressive surgical excision to prevent more invasion of the fungi. Despite that, neurological deficits persist including complete loss of vision in one eye which reflects the importance of early diagnosis and proper management to prevent fatal progression.

## Introduction

Mucormycosis is an angioinvasive fungal infection caused by filamentous molds within the order Mucorales [1]. It affects mainly immunocompromised patients and has a high mortality rate in immunocompromised patients, exceeding 30% to 50% [2]. The order Mucorales is a group of fungi classified under the class Zygomycetes. They are found in soil and decaying vegetation. The fungus is transmitted to humans by inhalation, ingestion, or cutaneous contact [3]. The median incubation period for the cutaneous pattern is one to two weeks, six days for ingestion, and two days for inhalational route [4]. As mucormycosis mostly affects immunocompromised people, the most common predisposing factor is diabetes mellitus (DM), and the number of patients with mucormycosis significantly increased [5,6].

There are five major clinical types of mucormycosis: rhinocerebral, pulmonary, cutaneous, gastrointestinal, and disseminated [7]. Other rare clinical types result in endocarditis, osteomyelitis, peritonitis, and renal infection [8]. The most reported type is rhinocerebral presenting 39% of the total cases, followed by pulmonary with 24% [9]. The primary diagnostic feature of invasive mucormycosis is tissue necrosis due to invasion of blood vessels and succeeding thrombosis. Useful tools for diagnosis are computed tomography, positron emission tomography-computed tomography (PET/CT) with (18F) fluorodeoxyglucose (FDG) [10], and magnetic resonance imaging (MRI) [11]. However, the gold standard test for early diagnosis is culture and histopathology of the clinical specimens from the infected site [12].

Mucormycosis can be effectively treated using appropriate antifungal medications, in combination with managing the predisposing risk factors such as ensuring proper glycemic control in patients with DM. Extensive surgical excision is a must to prevent fungal spreading, rapid deterioration, or death [13,14]. The available antifungal medications for mucormycosis include amphotericin B and its lipid formulations and triazoles such as posaconazole and isavuconazole [15]. Amphotericin B is the first drug approved by the FDA to treat mucormycosis by targeting ergosterol in the wall of the fungi, resulting in cell wall destabilization [15]. The dose depends on the affected area and the patient's condition [16]. Here, we report a case of rhino-orbital fungal mucormycosis associated with uncontrolled DM that followed the COVID-19 infection.

## Case presentation

We present a case of a 58-year-old male patient resident in Lahore who was recently diagnosed with DM. Glycated hemoglobin A1c (HbA1c) was 16.4, with no history of smoking or drug abuse, no family history related to any disease, and no history of surgery. There was no history of cardiac diseases or remarkable symptoms of other systems. The case was presented to the outpatient department (OPD) in the late period of 2021. The patient had a chief complaint of right eye ptosis and progressive painful deterioration of vision over 10 days' duration.

The condition started with a long course of mild to moderate COVID-19 infection for 15 days. The patient experienced mild shortness of breath, with their oxygen saturation levels remaining above 93% at all times. The disease was managed at home by systemic intravenous antibiotics and intravenous corticosteroids. After one week of recovery, the patient suffered from a sudden spontaneous nose bleeding. Four days later, he developed ocular symptoms in the following order: The patient initially presented with swelling around the right eye (periorbital edema). Three days later, they developed watering, redness, pain with eye movements, and mild forward protrusion (proptosis) of the right eye. Over the course of 7-8 days, the right eyelid gradually drooped (ptosis). As 10 days passed, the patient experienced a progressive decline in vision in the right eye, accompanied by restricted eye movements. During illness, the patient experienced remittent attacks of headache and dizziness.

The initial assessment of the eyes demonstrated significant ptosis and mild proptosis in the right eye, accompanied by restricted movement in all directions and a mid-dilated, nonreactive pupil. In contrast, the left eye showed normal adnexa, full extraocular movement, and a round, regular, reactive pupil. Further details are presented in Table 1.

**Table 1:** Initial assessment of the eyes

	Right eye	Left eye
Adnexa	Severe right eye ptosis mild proptosis	Unremarkable
Extraocular movements	Restricted in all directions	Full
Pupils	Mid dilated, fixed	Round, regular, reactive
Conjunctiva	Mild congestion	No congestion, chemosis
Lens	Nuclear sclerotic cataract +2, posterior subcapsular cataract grade 2	Nuclear sclerotic cataract +3, posterior subcapsular cataract grade 2
Cornea	Clear	Clear
Anterior chamber	Quiet, normal depth	Quiet, normal depth

**Figure 1:**
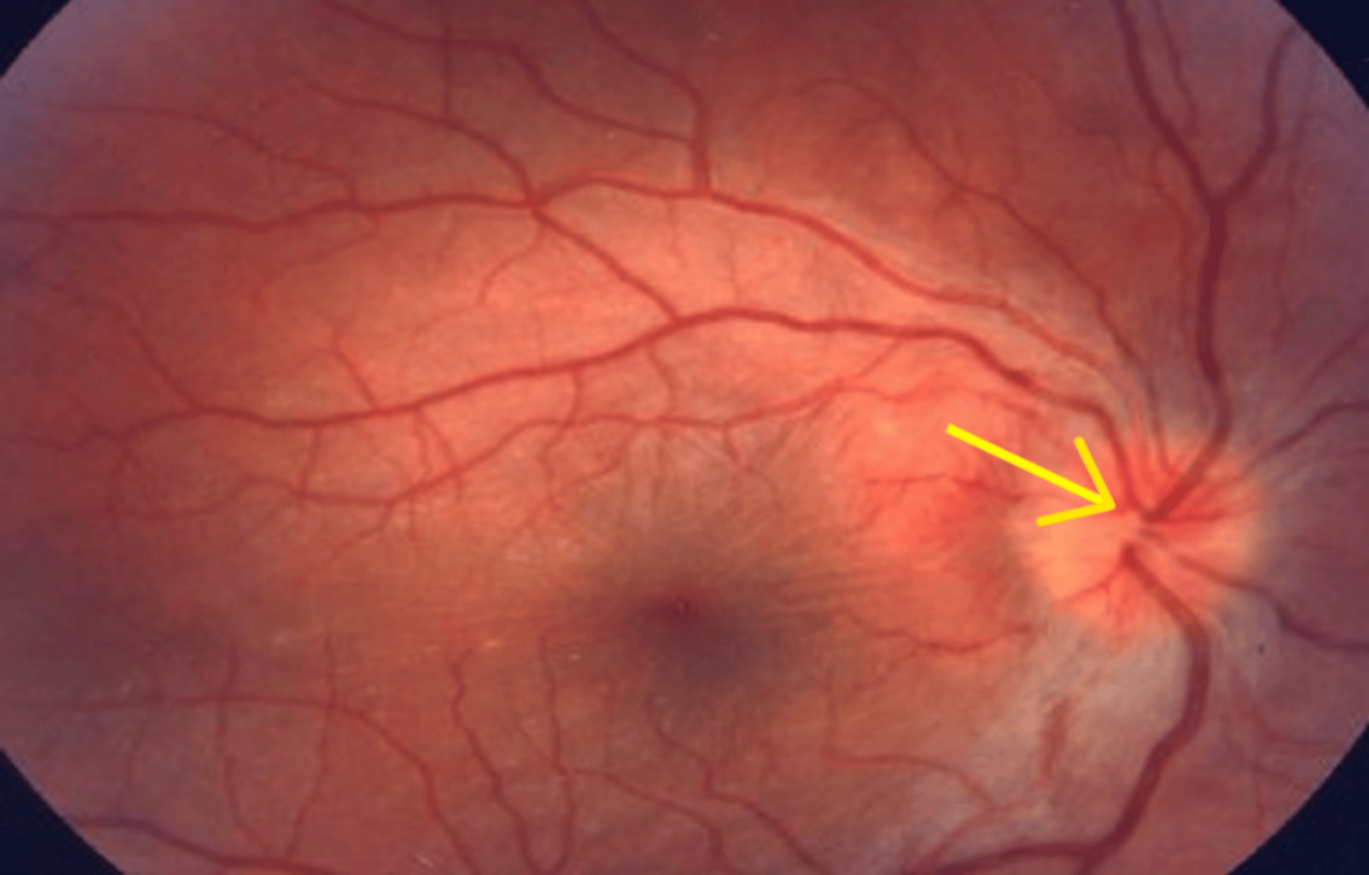
Fungus examination Blurred disc margins with obscuration of vessels inside and outside the optic nerve head

A comprehensive ocular evaluation was conducted, including a clinical examination along with the assessment of visual acuity, intraocular pressure, and a detailed fundus examination, showing papilledema as there’s blurring of the optic disc margin and a cup-to-disc ratio CD ratio cannot be identified here as shown in Figure 1.

A detailed fundus examination was performed to evaluate the posterior segment of both eyes. The optic disc appeared hyperemic in the right eye, and the CD ratio was indeterminate. However, the macula was grossly healthy. As the disease progressed, the optic disc developed pallor. In contrast, the left eye examination revealed an unremarkable optic disc with a CD ratio of 0.3. The retinal vasculature was intact, and the macula remained grossly healthy.

In examining the visual acuity and color vision, the right eye exhibited no light perception, indicating total visual loss. The left eye demonstrated an uncorrected visual acuity (UCVA) of 6/24, improving to a best corrected visual acuity (BCVA) of 6/9 with optical correction. Color vision in the left eye was found to be normal. The intraocular pressure measured 10 mmHg in the right eye and 12 mmHg in the left eye. There was a mild proptosis of the right eye, and Hertel’s exophthalmometer measurements were 23 mm and 19 mm in the right and left eye, respectively.

A physical examination was conducted to assess ptosis, revealing vertical palpebral fissure heights of 4 mm in the right eye and 11 mm in the left eye. Additional findings are documented in Table 2.

**Table 2:** Ptosis evaluation

	Right eye	Left eye
Vertical fissure height	4 mm	11 mm
Margin reflex distance	Could not be measured because the lid was covering the pupillary axis	4 mm
Upper lid crease (lid margin and lid crease in downward gaze)	8 mm	10 mm
Pretarsal show ( lash line and skinfold in the primary position of gaze)	4 mm	3 mm
Levator function	6 mm	13 mm

Laboratory investigations were performed, including a complete blood count, liver function tests, renal function tests, serum electrolytes, complete urine examination, erythrocyte sedimentation rate (ESR), C-reactive protein (CRP), and viral serologies for hepatitis B virus (HBV), hepatitis C virus (HCV), and human immunodeficiency virus (HIV). Moreover, fasting and random blood glucose levels, as well as HbA1c, were assessed. The results indicated an elevated CRP level of 25.65 mg/dL and a significantly high HbA1c level of 16.3%. Random blood glucose was measured at 417 mg/dL, while the fasting blood glucose level was 303 mg/dL. Laboratory investigations are shown in a simple form in Table 3.

**Table 3:** Laboratory investigations CRP: C-reactive protein; HbA1c: glycated hemoglobin A1c

Parameter	Result	Normal range
HbA1c	16.3%	<5.7%
Random blood glucose	417 mg/dL	<140 mg/dL
Fasting blood glucose	303 mg/dL	<100 mg/dL
CRP	25.65 mg/dL	<3.0 mg/dL

**Figure 2:**
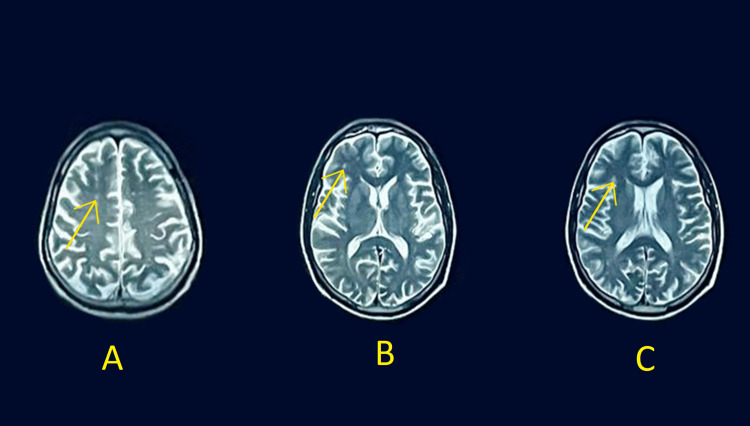
MRI of the brain and orbit MRI: magnetic resonance imaging Different levels of MRI labeled from A to C that show chronic microvascular ischemic cerebral infarcts in the right frontal region

MRI of the brain and orbit demonstrated the presence of chronic microvascular ischemic infarcts in the right frontal region. There was extensive mucosal thickening observed in the bilateral frontal, ethmoid, and sphenoid sinuses, along with mild mucosal thickening in the right maxillary sinus. Additionally, mild mastoiditis was noted on the right side, and there was minimal fluid accumulation surrounding the right optic nerve (Figure 2).

**Figure 3:**
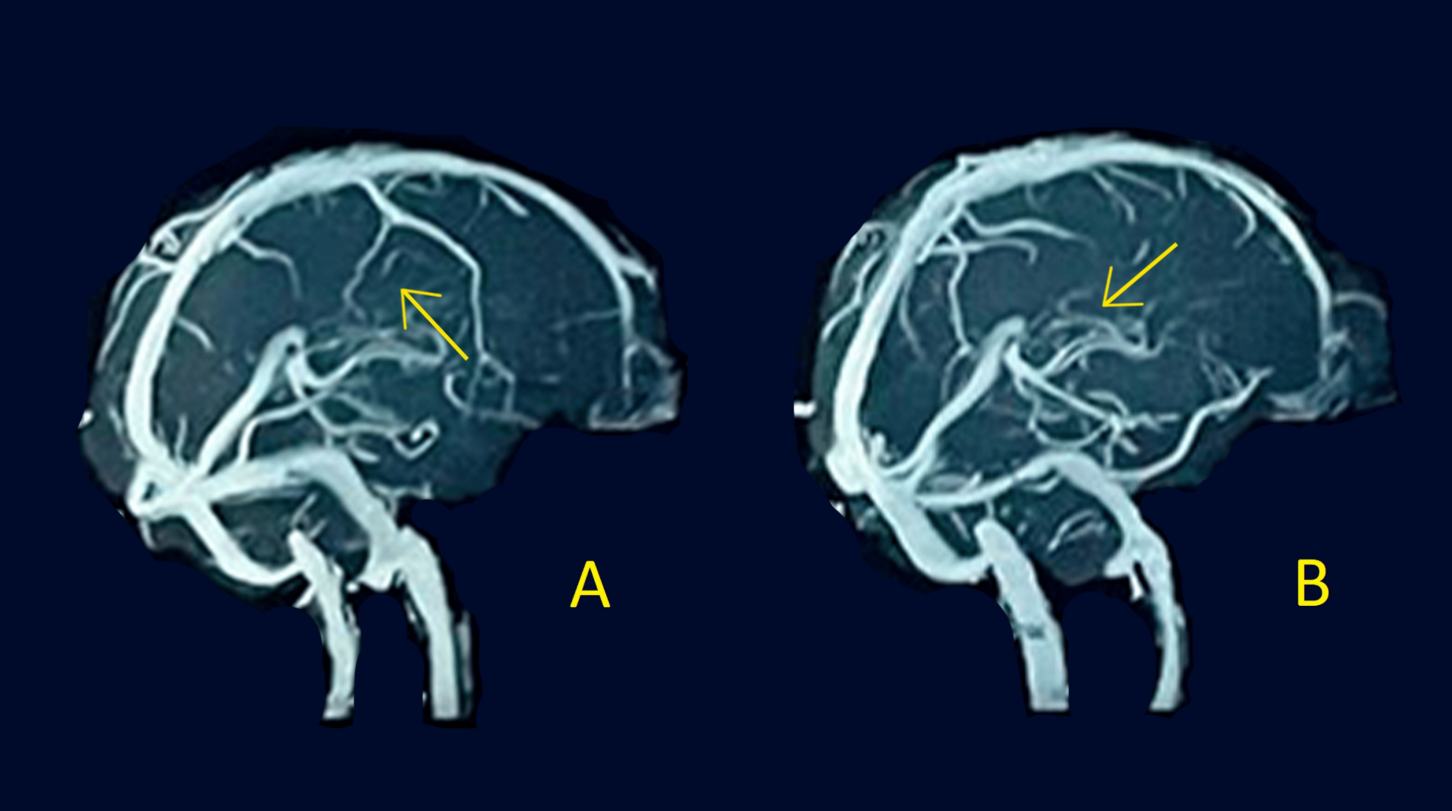
MRV of the brain and orbit MRV: magnetic resonance venography Two different figures: A and B

A further MR venography (MRV) of the brain and orbit with contrast was performed, revealing mild retrobulbar fat stranding in the apex region on the right side. There was also a mild enlargement of the extraocular muscles and mild prominence of the cerebrospinal fluid (CSF) sleeve surrounding the right optic nerve. In addition, a mild hyperintense signal was noted within the optic nerve at the orbital apex. Furthermore, moderate rhinosinusitis was observed, characterized by a right-sided stomatal and spheno-ethmoidal pattern. MRV of the brain and orbit is shown in Figure 3.

A culture tissue biopsy was obtained from the nasal cavities and sinuses, and microscopic examination revealed polypoidal masses lined by benign columnar epithelium. Broad, aseptate branching hyphae were observed, and special stains for fungi tested positive. The microscopic diagnosis was inflammatory nasal polyps with mucormycosis. Based on our histopathological and radiological evidence, the final diagnosis was sino-orbital mucormycosis with orbital apex syndrome.

The patient was admitted to the ward, and insulin was initiated immediately. Debridement, right lateral rhinotomy, and endoscopic clearance with medial maxillectomy were performed to achieve proper eradication. The systemic medical treatment was conducted, including liposomal amphotericin B 200 mg once daily for eight weeks, ceftriaxone 1 g twice daily for 14 days, and Provas 2 g as needed. In addition, topical treatments were administered, including tobramycin eye ointment at bedtime and moxifloxacin eye drops three times daily for a week. Nine days after surgery, proptosis and painful aye movement resolved, but all other cranial nerve symptoms, such as restricted eye movement, ptosis, and no light perception (NLP) in the right eye, are still present.

## Discussion

In most cases, mucormycosis is a rare, opportunistic, life-threatening fungal infection. However, we have seen a sudden increase in mucormycosis cases after the COVID-19 pandemic. Mucormycosis is the third most common invasive fungal infection following aspergillosis and candidiasis [17].

Most cases infected with mucormycosis were from India. According to the minister of Maharashtra, approximately 2000 cases were reported in the state till May 11, 2021 [18]. Around 47,000 cases were reported in India from May to July 2021 [19]. Rhino-orbital and rhino-orbito-cerebral were the most common presentations [19]. Most of the patients were recently treated for COVID-19 and diabetics with poorly controlled hyperglycemia. It is believed that the excessive use of corticosteroids especially when treating COVID-19 is the cause [20], while others believe that the virus is immunosuppressive enough to help the fungus spread [21].

Various risk factors may result in mucormycosis, and the most common risk factor is DM [5,6]. Other risk factors are hematological malignancies and hematological conditions such as aplastic anemia [22]. Other predisposing factors include solid organ malignancies, organ transplantation, neutropenia (WBCs < 1500/mm^3^), corticosteroids, autoimmune diseases (such as systemic lupus erythematosus (SLE)), HIV, trauma, and recent COVID-19 infection [5,23]. However, a meta-analysis, published in 2022 including 693 patients, found that 275 patients (39.6%) had no underlying disease or predisposing condition [22]. In our presented case, the patient was diabetic and was on corticosteroids for a long period.

DM disrupts the innate immune system by impairing phagocytic activity and delaying the activation of adaptive immune responses due to compromised dendritic cell function. In addition, COVID-19 can exacerbate DM and develop diabetic ketoacidosis [24,25]. Moreover, certain pathophysiological characteristics of COVID-19 may increase the risk of secondary fungal infections. SARS-CoV-2 infection is marked by immune dysregulation, including T-cell lymphopenia and impairment of innate immunity [24].

Thrombotic events leading to vascular endothelial damage may also enhance fungal invasion, alongside elevated ferritin and iron levels, activation of hepcidin, and upregulation of GRP78 receptors [25,26]. High-dose steroids were found to be used by most patients with COVID-19-associated mucormycosis [26,27]. Steroids induce both immunosuppression and muscle catabolism and atrophy [26]. Most reported cases of COVID-19-associated mucormycosis (CAM) have occurred during active SARS-CoV-2 infection. However, several studies documented that CAM cases occurred during the recovery phase which occurred in our case [25].

In a multicenter epidemiological study by Patel et al. [28], the majority of mucormycosis cases associated with COVID-19 were found to be rhino-orbital, accounting for 62.6% of cases, followed by the rhino-orbito-cerebral form in 23.5% of cases, and the pulmonary form in 8.6%.

In advanced cases of rhino-orbital or rhinocerebral disease, patients may develop trigeminal or other cranial nerve palsies. The infection can rapidly progress to involve the central nervous system, potentially leading to carotid artery thrombosis. A bloody nasal discharge may be the only indication of infection spreading from the nasal turbinates to the brain [5]. Due to the unspecific symptoms and signs, diagnosis of mucormycosis needs high clinical suspicion, acknowledgment of host factors, and immediate clinical assessment [5,12]. Periorbital swelling, sinus pain, proptosis, cranial nerve palsy, orbital apex syndrome, and palate ulcers are all warning signs and symptoms and need prompt and optimum performance [29].

Our case was a typical case of rhino-orbital mucormycosis. The patient had an uncontrolled DM and had recently recovered from COVID-19 which was treated with systemic corticosteroid. Each of them is considered a significant risk factor for mucormycosis. In addition, he presented to the OPD with typical symptoms of orbital infection, right eye ptosis, proptosis, preorbital cellulitis, and orbital apex syndrome. All these symptoms are considered indicators and warnings in such a case. Furthermore, symptoms of sinus infection include nosebleeds. The definite diagnosis was established by detecting broad aseptate branching hyphae and also by using special stains for fungus which were positive. Once the patient was diagnosed with mucormycosis, we started to control DM with insulin, along with amphotericin B which is the first-line antifungal for mucormycosis. Proper surgical excision was performed to prevent progression.

## Conclusions

We focused on the risk of secondary fungal infections, particularly mucormycosis, in COVID-19-recovering patients with uncontrolled DM. High clinical suspicion is crucial for early detection in immunocompromised patients, especially those with uncontrolled DM and recent COVID-19. Delayed diagnosis and treatment can lead to aggressive progression and poor outcomes. Proper management of underlying risk factors, such as DM, and minimizing corticosteroid use in COVID-19 patients with DM can reduce the risk of secondary mucormycosis. Effective management requires coordination between various specialties for early diagnosis and proper treatment to avoid complications, enhance survival, and limit morbidity.
